# MECP2 duplication syndrome—Typical EEG characteristics

**DOI:** 10.1002/epd2.70015

**Published:** 2025-04-01

**Authors:** Walter Otu, Ritu Sudhakaran, German Garza‐Garcia, Krishna Parekh, Irfan S. Sheikh

**Affiliations:** ^1^ University of Texas Southwestern Medical Center Dallas Texas USA; ^2^ Division of Epilepsy, Department of Neurology University of Texas Southwestern Medical Center Dallas Texas USA; ^3^ Peter O'Donnell Jr. Brain Institute University of Texas Southwestern Medical Center Dallas Texas USA

We report a 36‐year‐old male with a history of psychomotor and intellectual delay, right hemiparesis, and seizures since age 17 controlled on valproic acid, felbamate, and rufinamide. Chromosomal microarray performed at age 28 revealed a copy number gain of chromosome band Xq28 (3.273 Mb in size), including the MECP2 gene, consistent with MECP2 duplication syndrome (MDS).^1^ EEG demonstrated a poorly organized background with generalized delta slowing and bilateral independent frontotemporal interictal epileptiform discharges (Figure 1). EEG findings in patients with MDS can be consistent with generalized delta slowing and interictal findings of generalized slow spike‐andand‐wave asynchronous discharges with frontotemporal predominance. Additional EEG findings can include interictal sharp and slow wave discharges with frontotemporal predominance and generalized paroxysmal fast activity (GPFA).^2^, ^3^, ^4^ This patient's history is additionally notable for recurrent infections and autistic features, which are other common manifestations of MDS.^5^, ^6^

**FIGURE 1:**
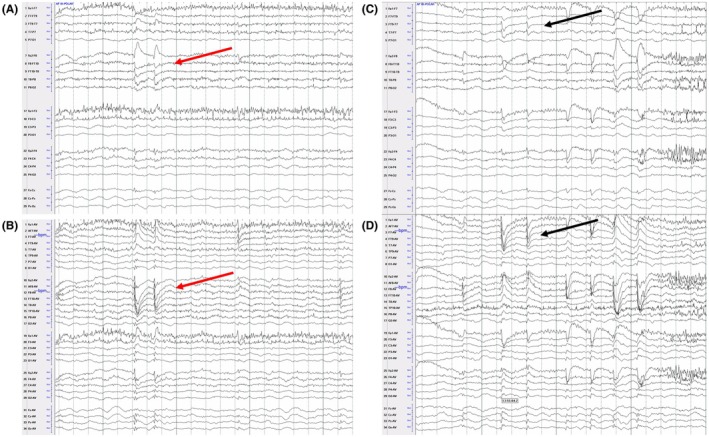
EEG of MECP2 duplication syndrome. (A, B) Bipolar and average montage of EEG showing generalized delta slowing and right frontotemporal epileptiform discharges with a broad right hemispheric field (red arrow). (C, D) Bipolar and average montage EEG showing generalized delta slowing and left frontotemporal epileptiform discharges (black arrow).

**Test yourself**

1. Which EEG pattern can be seen in patients with MECP2 duplication syndrome?
  - Normal background activity
  - Generalized slowing with focal/multifocal discharges
  - Continuous spike‐and‐wave during sleep
  - Hypsarrhythmia
2. Which of the following is a common comorbidity in MECP2 duplication syndrome?
  - Cardiomyopathy
  - Recurrent respiratory infections
  - Renal failure
  - Diabetes mellitus
3. What is the inheritance pattern of MECP2 duplication syndrome?
  - Autosomal dominant
  - Autosomal recessive
  - X‐linked recessive
  - Mitochondrial

*Answers may be found in the* *supporting information*

## Supporting information


Data S1.

Data S2.

## Data Availability

The data that support the findings of this study are available on request from the corresponding author. The data are not publicly available due to privacy or ethical restrictions.
