# Supplementary material for: MECP2 duplication syndrome—Typical EEG characteristics
Source: Epileptic Disord. 2025 Apr 1;27(3):487–8. doi: 10.1002/epd2.70015 (PMC12203300; doi:10.1002/epd2.70015)
Supplement: Supplementary file 1 — Data S1. [file EPD2-27-487-s001.pptx]

## Slide 1
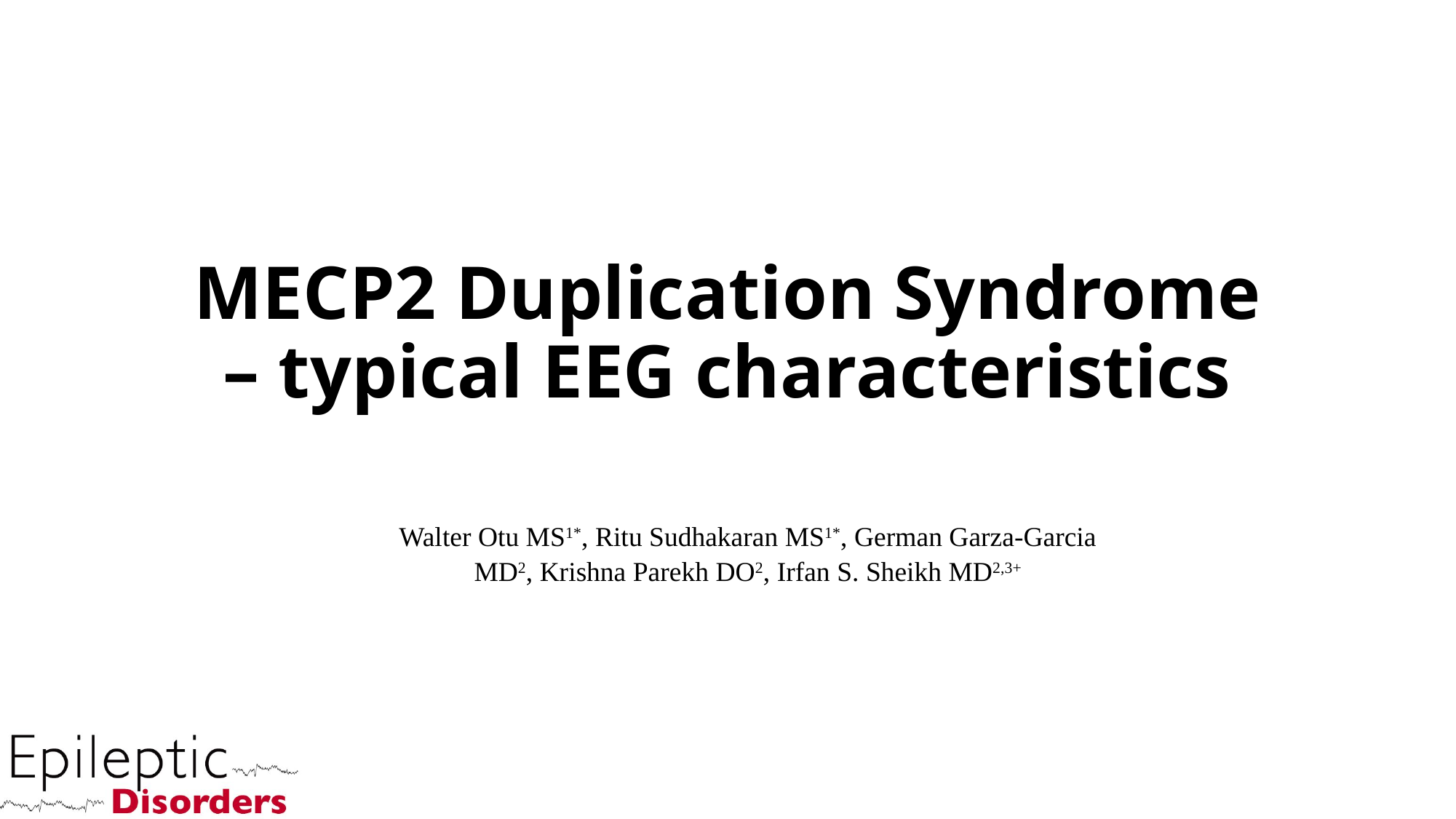

# MECP2 Duplication Syndrome – typical EEG characteristics
Walter Otu MS1*, Ritu Sudhakaran MS1*, German Garza-Garcia MD2, Krishna Parekh DO2, Irfan S. Sheikh MD2,3+

## Slide 2
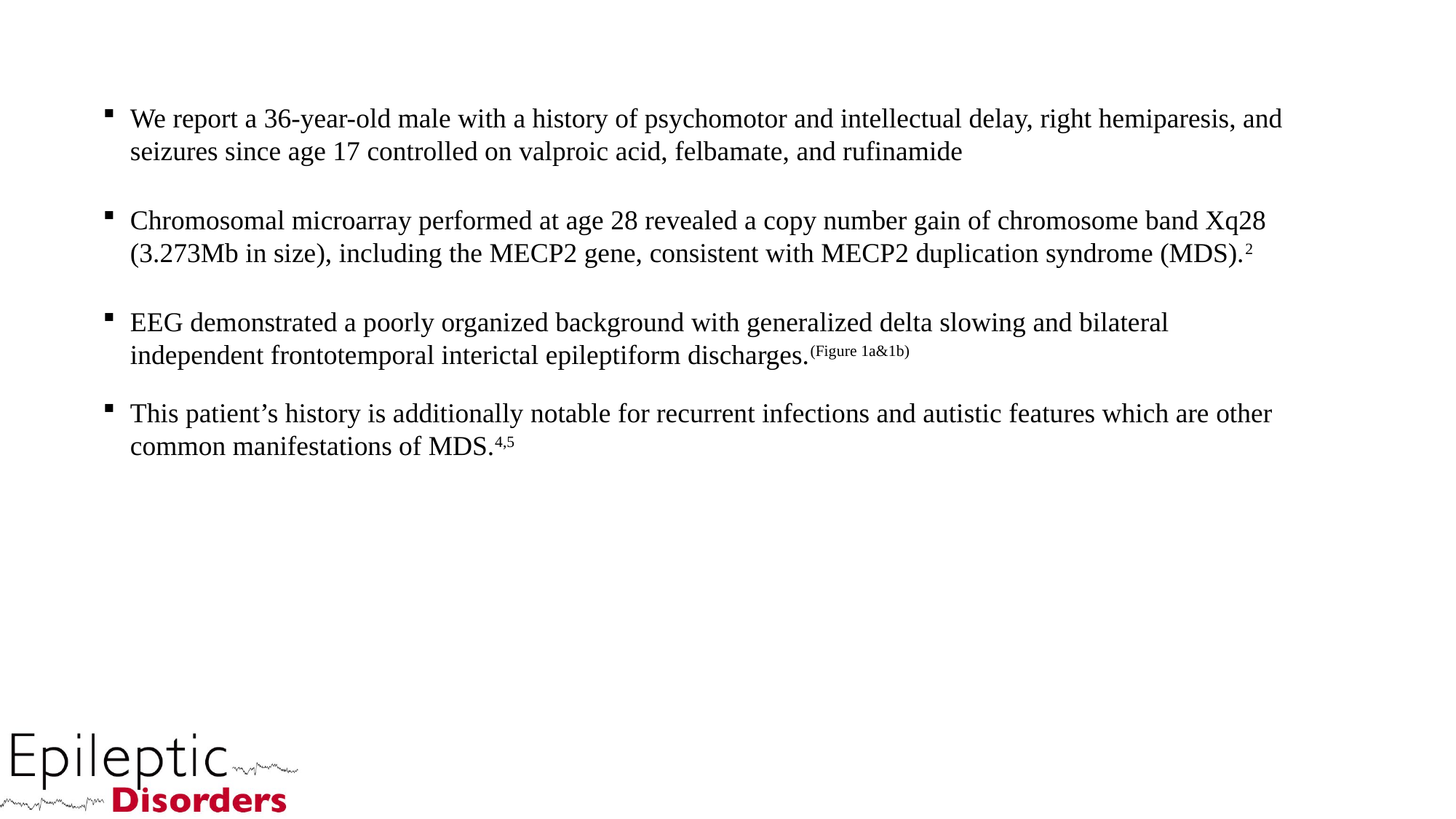

We report a 36-year-old male with a history of psychomotor and intellectual delay, right hemiparesis, and seizures since age 17 controlled on valproic acid, felbamate, and rufinamide
Chromosomal microarray performed at age 28 revealed a copy number gain of chromosome band Xq28 (3.273Mb in size), including the MECP2 gene, consistent with MECP2 duplication syndrome (MDS).2
EEG demonstrated a poorly organized background with generalized delta slowing and bilateral independent frontotemporal interictal epileptiform discharges.(Figure 1a&1b)
This patient’s history is additionally notable for recurrent infections and autistic features which are other common manifestations of MDS.4,5

## Slide 3
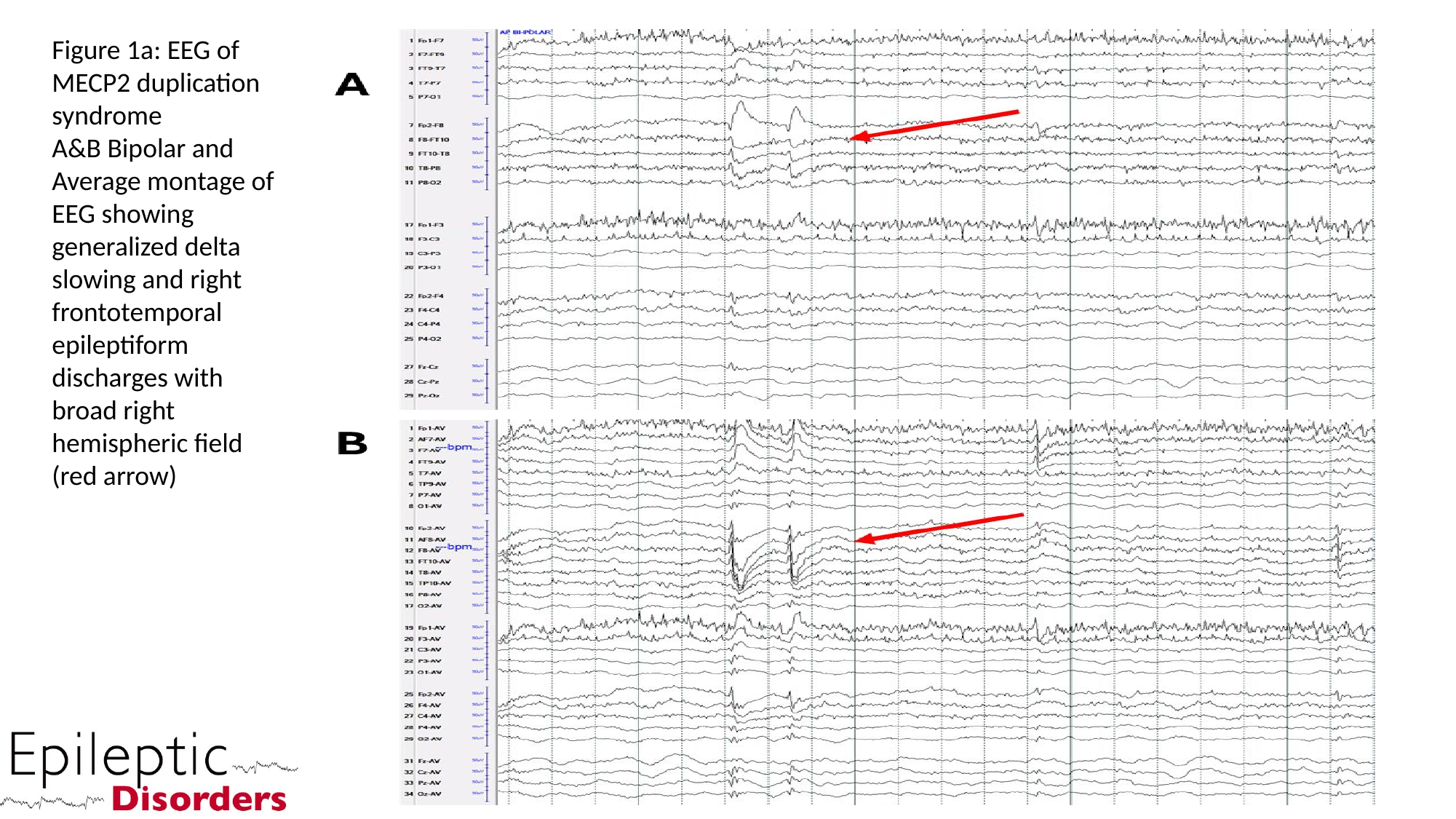

Figure 1a: EEG of MECP2 duplication syndrome
A&B Bipolar and Average montage of EEG showing generalized delta slowing and right frontotemporal epileptiform discharges with broad right hemispheric field (red arrow)

## Slide 4
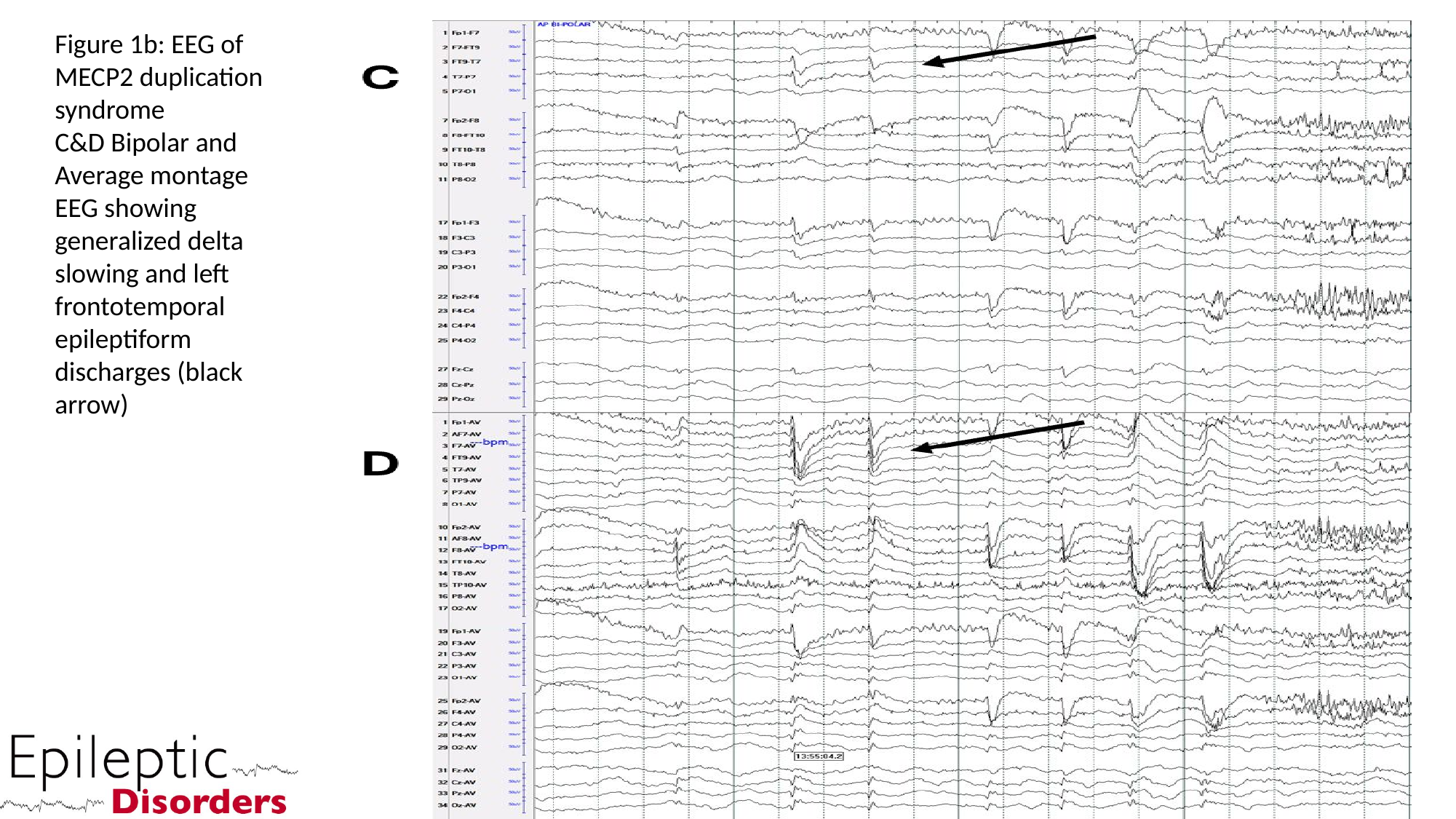

Figure 1b: EEG of MECP2 duplication syndrome
C&D Bipolar and Average montage EEG showing generalized delta slowing and left frontotemporal epileptiform discharges (black arrow)

## Slide 5
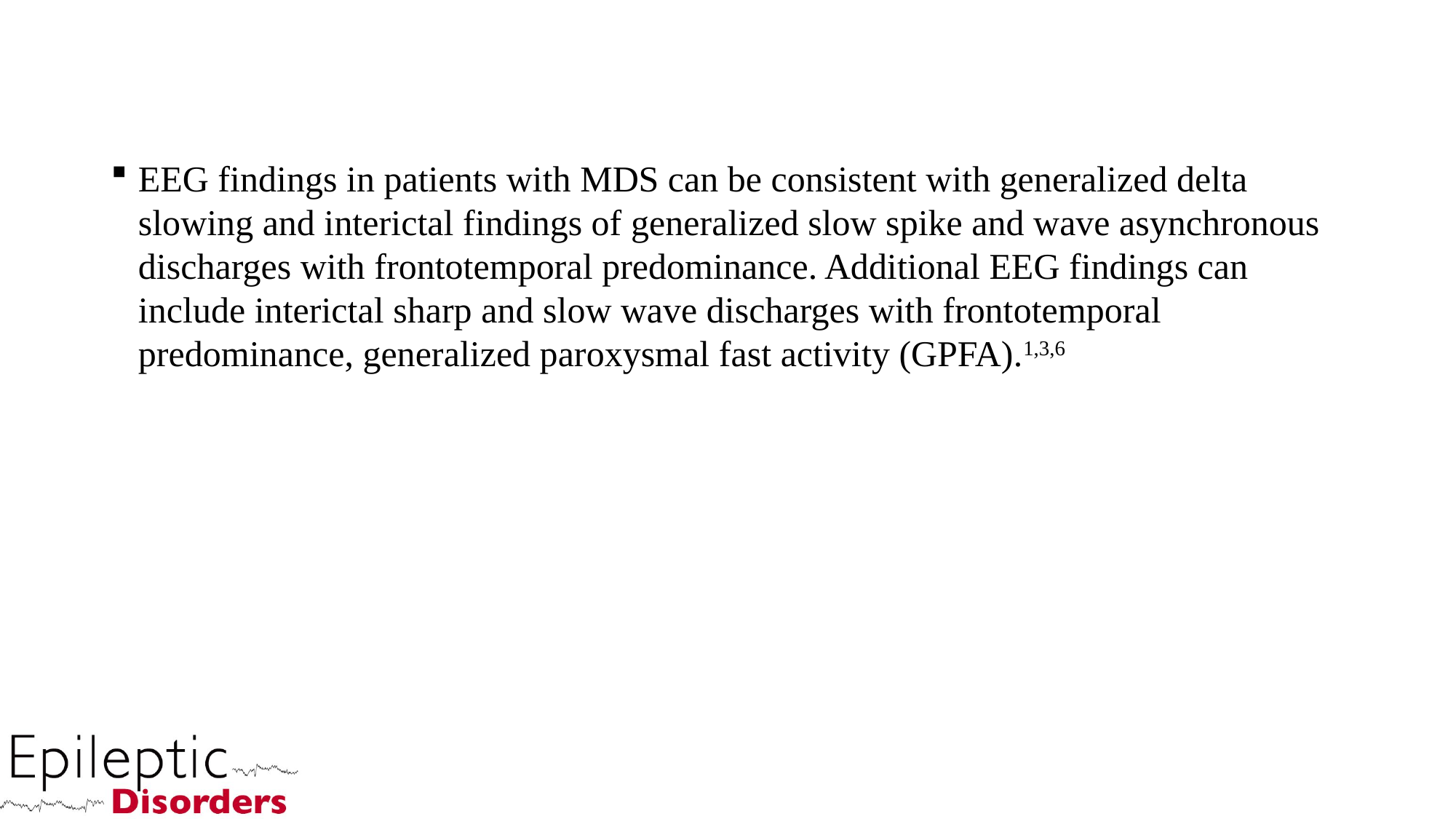

EEG findings in patients with MDS can be consistent with generalized delta slowing and interictal findings of generalized slow spike and wave asynchronous discharges with frontotemporal predominance. Additional EEG findings can include interictal sharp and slow wave discharges with frontotemporal predominance, generalized paroxysmal fast activity (GPFA).1,3,6

## Slide 6
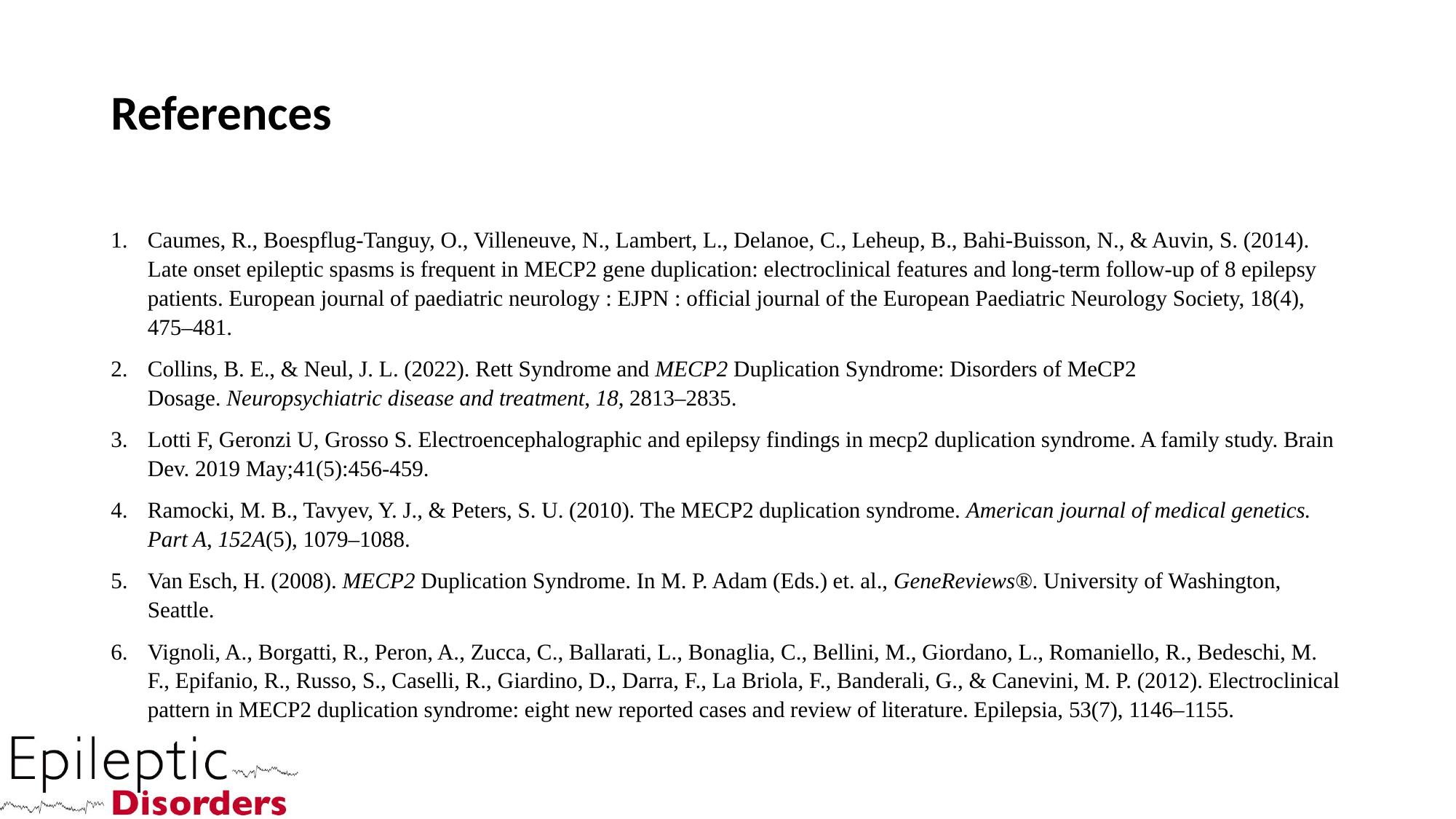

# References
Caumes, R., Boespflug-Tanguy, O., Villeneuve, N., Lambert, L., Delanoe, C., Leheup, B., Bahi-Buisson, N., & Auvin, S. (2014). Late onset epileptic spasms is frequent in MECP2 gene duplication: electroclinical features and long-term follow-up of 8 epilepsy patients. European journal of paediatric neurology : EJPN : official journal of the European Paediatric Neurology Society, 18(4), 475–481.
Collins, B. E., & Neul, J. L. (2022). Rett Syndrome and MECP2 Duplication Syndrome: Disorders of MeCP2 Dosage. Neuropsychiatric disease and treatment, 18, 2813–2835.
Lotti F, Geronzi U, Grosso S. Electroencephalographic and epilepsy findings in mecp2 duplication syndrome. A family study. Brain Dev. 2019 May;41(5):456-459.
Ramocki, M. B., Tavyev, Y. J., & Peters, S. U. (2010). The MECP2 duplication syndrome. American journal of medical genetics. Part A, 152A(5), 1079–1088.
Van Esch, H. (2008). MECP2 Duplication Syndrome. In M. P. Adam (Eds.) et. al., GeneReviews®. University of Washington, Seattle.
Vignoli, A., Borgatti, R., Peron, A., Zucca, C., Ballarati, L., Bonaglia, C., Bellini, M., Giordano, L., Romaniello, R., Bedeschi, M. F., Epifanio, R., Russo, S., Caselli, R., Giardino, D., Darra, F., La Briola, F., Banderali, G., & Canevini, M. P. (2012). Electroclinical pattern in MECP2 duplication syndrome: eight new reported cases and review of literature. Epilepsia, 53(7), 1146–1155.
