# Supplementary material for: MECP2 duplication syndrome—Typical EEG characteristics
Source: Epileptic Disord. 2025 Apr 1;27(3):487–8. doi: 10.1002/epd2.70015 (PMC12203300; doi:10.1002/epd2.70015)
Supplement: Supplementary file 2 — Data S2. [file EPD2-27-487-s002.docx]

1. Answer: B

2. Answer: B

3. Answer: C
